# Comparison of patients’ oral health-related quality of life and chewing efficiency between conventional and 3D-printed complete dentures: A crossover clinical trial

**DOI:** 10.34172/joddd.025.44430

**Published:** 2025-12-31

**Authors:** Radwa Mohsen Kamal Emera, Zeena Farhan AL Sultani, Fatma Fathe Mahanna

**Affiliations:** ^1^Department of Removable Prosthodontics, Faculty of Dentistry, Mansoura University, Egypt; ^2^Department of Conservative Treatment, College of Dentistry, Alqadisiyah University, Aldiwaniya, Iraq

**Keywords:** 3D printing, CAD/CAM, Chewing efficiency, Complete removable dentures, Digital dentures, Oral health-related quality of life

## Abstract

**Background.:**

Additive manufacturing has introduced novel materials and workflows for complete denture fabrication; however, high-quality clinical evidence comparing patient-centered outcomes of 3D-printed and conventionally fabricated dentures remains limited. This compared oral health-related quality of life (OHRQoL) and chewing efficiency between conventional and 3D-printed dimethacrylate-based resin complete dentures.

**Methods.:**

Twenty completely edentulous patients were enrolled in this randomized two-period crossover clinical trial. Each participant received both interventions—conventional complete dentures and 3D-printed dimethacrylate-based resin complete dentures—in a randomized sequence. OHRQoL was assessed using the Oral Health Impact Profile for Edentulous Patients (OHIP-EDENT), and chewing efficiency was evaluated using a two-colored chewing gum mixing ability test by calculating the standard deviation of hue (H-SD). Assessments were performed at insertion (T_0_), after 3 months (T_3_), and after 6 months (T_6_). Normality was assessed using the Shapiro–Wilk test. Intra-subject comparisons were conducted using the Wilcoxon signed-rank test for OHIP-EDENT scores and paired t-tests for chewing efficiency. Statistical significance was set at *P*≤0.05.

**Results.:**

No statistically significant differences were observed between conventional and 3D-printed dentures regarding overall OHIP-EDENT scores or individual domains at T_0_, T_3_, or T_6_ (*P*>0.05). Both denture types demonstrated significant improvements in OHRQoL from the pre-treatment period to T_6_ (*P*<0.05). Similarly, chewing efficiency showed no significant differences between denture types across all chewing cycles (*P*>0.05).

**Conclusion.:**

Within the limitations of this randomized crossover study, 3D-printed dimethacrylate-based resin complete dentures demonstrated clinical performance and satisfaction comparable to conventional dentures, supporting their use as a reliable alternative in complete denture rehabilitation.

## Graphical Abstract

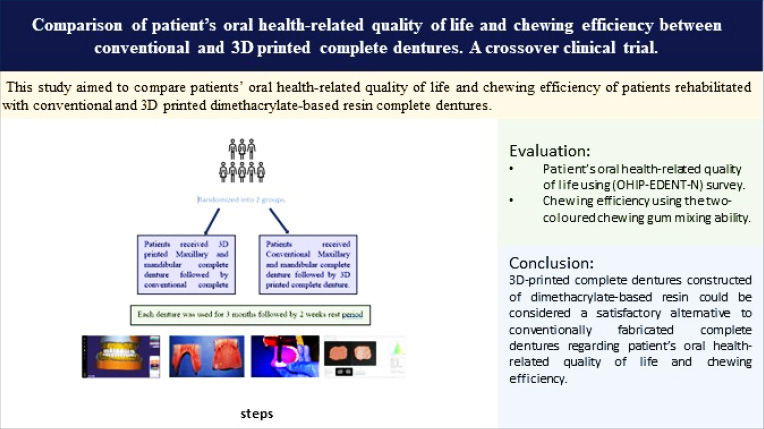

## Introduction

 Edentulism—complete tooth loss—has long been a global public health issue. It impairs elderly individuals’ speech and masticatory efficacy. Poor oral and overall health, nutritional status, and quality of life are common among elderly edentulous patients.^1^ For edentulous patients with physical, psychological, or economic constraints that prevent implant therapy, the most common prosthetic treatment is rehabilitation with a complete removable dental prosthesis.^2^

 The traditional method for producing complete removable dental prostheses has been in use for more than eight decades. Since then, it has been relatively stable, effective, and dependable.^3^ Conventional complete removable dental prosthesis manufacture, however, requires a series of clinical and manual laboratory procedures. Conventional denture production uses heat-cured polymethylmethacrylate (PMMA) materials, which can cause issues such as 1) polymerization shrinkage that reduces the fit between denture-bearing tissues and denture base; 2) lack of dimensional stability; 3) increased residual monomers; 4) water absorption; 5) color changeability; 6) difficulty in duplicating dentures; and 7) denture porosity, which affects denture aesthetics and mechanics. *Candida albicans* can also be found under the denture, increasing the risk of infection. Many conventional complete denture wearers are also dissatisfied.^4,5^ Most patients complain about prosthesis instability, retention, and speech and chewing problems.^6^

 For the construction of complete dentures, computer-aided design and computer-aided manufacturing approaches have been developed to address issues associated with conventional removable complete dental prostheses. CAD/CAM manufacturing of complete removable dental prostheses is performed mostly using additive manufacturing, 3D printing (also known as rapid prototyping [RP]), or computer numerical control (CNC) machining.^7^

 By joining materials layer by layer, as in additive manufacturing or 3D printing, models can be created from digital 3D data. Charles Hull pioneered the application of this technique in the late 1980s, and since the 1990s, it has been used to create anatomical 3D models for surgery. Subcategories of additive manufacturing rely on the materials and processes employed. The dentistry industry frequently uses stereolithography tools (SLA) and selective laser sintering (SLS). The use of UV polymerizable resins in the SLA technique has led to extensive use in the creation of dental restorations.^7,8^

 RP approaches have a collective advantage over CAD-CAM milling processes. Milling units are costly and may be appropriate for commercial manufacturing facilities, but they are impractical for individual practices and smaller dental laboratories. Furthermore, producing these units requires a large amount of energy. In addition, the subtractive manufacturing technique of milling results in substantial material waste.^9^

 A review of the literature revealed little information on the materials used for 3D-printed complete dentures; therefore, this study aimed to investigate 3D-printed complete dentures as a denture base material for patients’ oral health-related quality of life and chewing efficiency. The null hypothesis was that there was no significant difference in patients’ oral health-related quality of life and chewing efficiency between the two prostheses.

## Methods

 Twenty patients (12 males and 8 females), aged 55‒75 years, were selected from the Prosthodontic Department Clinic, Faculty of Dentistry. The patients were selected based on the absence of systemic disorders that influence bone metabolism, such as uncontrolled diabetes or osteoporosis, and on their good health. This was accomplished through a thorough medical history and clinical examination conducted by a physician. Before this, they had no previous dentures. The residual alveolar ridges were covered with healthy, firm mucosa, with Angle’s Class I maxillary‒mandibular relationship. The patients were fully informed about the purpose and the procedures of this study and signed written consent. The trial received approval from the Ethics Committee of the Faculty(approval no.: A04080120) and was registered at ClinicalTrials.gov (ClinicalTrials.gov Identifier: NCT06103019 (26/10/2023)

###  Study Design 

 The trial had a crossover design. Each patient received two different types of prostheses. This design allowed for the standardization of intra-patient variables that could affect the evaluation. In addition, each patient served as their own control.

 Each patient received two prostheses: 1) conventional maxillary and mandibular complete dentures; 2) 3D-printed complete maxillary and mandibular dentures. To minimize the consequences of the order of complete denture insertion on patients’ oral health-related quality of life outcomes, the sequence of insertion was randomized. Using random numbers in a Microsoft Excel spreadsheet, the participants were randomly assigned to two groups. The randomization data were generated by a dentist who was blinded to the type of restoration. Each denture was worn for 3 months, followed by a 2-week rest period without dentures, and then the other type of denture was worn for another 3 months. The participant’s flow diagram is presented in Supplementary file.

###  Sample Size Calculation

 The sample size was determined based on the results of a clinical trial 10 that revealed a significant difference in chewing efficiency between conventional and 3D-printed complete dentures (α = 0.05, β = 0.15). The calculated sample size (17 patients) was increased to 20 to account for potential dropouts. The power analysis was performed using computer software (G*power 3.1.5).

###  Prosthetic Procedures

 For each patient, conventional maxillary and mandibular complete dentures were constructed, followed by 3D-printed complete denture construction as follows: Using a 3D scanner, complete maxillary and mandibular dentures as well as master casts were scanned (VDSL Home Gateway model EchoLife DG8045 China) after applying a thin layer of antiglare spray (Siladent MarmoScan spray basic). Using specialized software (Exocad DentalIDB 2.4 plovdiv7290 [version 2.4 Enginebuild 7290]), the data were exported to the CAD-CAM complete denture manufacturer in STL format. The virtual model was subsequently used to construct the definitive complete denture, with the anatomic landmarks recognized and the peripheral borders marked.

**Figure 1:**
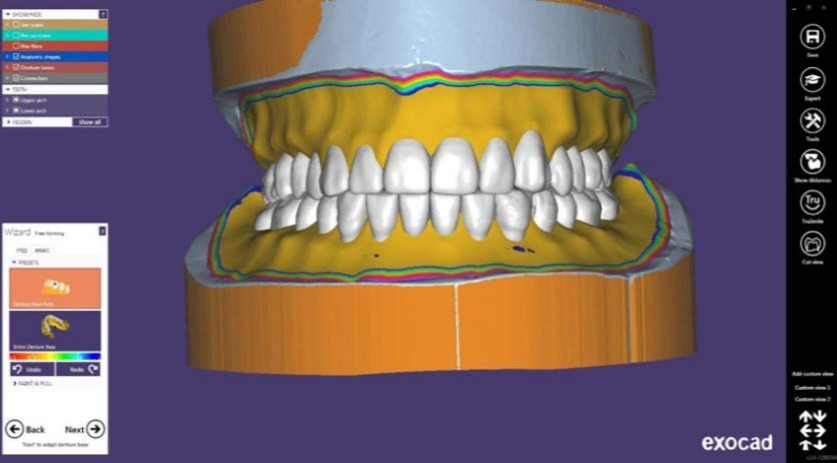
3D image of the superimposition of scanned conventional dentures on 3D-designed dentures

 The STL image of the scanned conventional complete denture was superimposed on the designed denture for comparison of the complete denture’s polished surface, tooth size, shape, and alignment (Figure 1). Before fabrication, a digital preview was developed and sent for approval.

###  Printing Preparation 

 The cassette of the RASDEN 3D printer was filled with the selected shade of DENTCA Denture Base II (a dimethacrylate-based resin with a photoinitiator). The printer door subsequently closed.

###  Printing Procedures 

**Figure 2:**
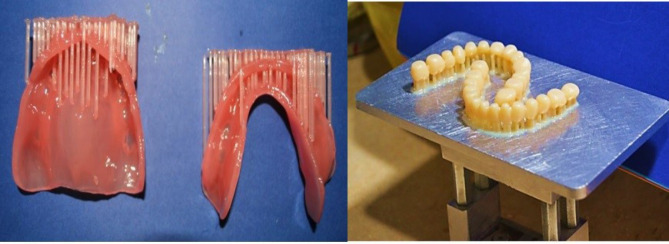
Printed denture bases and teeth with supporting posts

 The STL file of the denture base model was uploaded to the software. The denture base was vertically positioned on the build platform. Then, the denture base was reinforced with support. After the desired slice thickness was selected, printing of the denture base was initiated. Teeth were printed as one unit (Dentca denture teeth, shade A2) (Figure 2).

###  Cleaning

 The printed denture base and teeth were removed from the build platform, and the remaining supports were removed. The denture base was cleaned with isopropyl alcohol (IPA). The container of denture base/teeth and IPA was placed in a water bath with an ultrasonic vibratorfor shaking and cleaning. The denture base and teeth were dried with paper towels before curing.

###  Attaching Denture Teeth to The Base 

**Figure 3:**
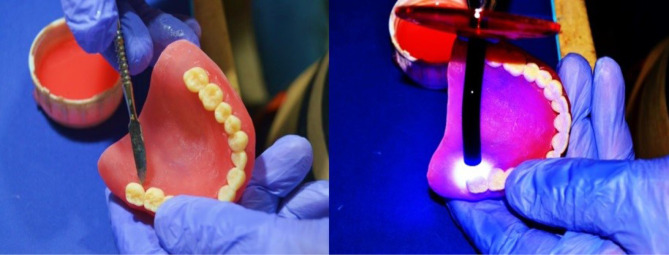
Bonding of the teeth to the denture base

 A small quantity of light-curing, shade-matched adhesive was used to bond the printed teeth to the printed denture base into the corresponding tooth sockets(Figure 3). The support spots on the denture base were smoothed using a bur or hand tool.

###  Postcuring Procedures

 The final denture was cured by complete submergence in a glycerol container for a) 20 min in Dymax ECE 5000, after which the dentures were flipped halfway through the post-cure; b) 30 min under vacuum at 90% LED intensity in a Dreve PCU LED with no flipping (both sides cured simultaneously), and the glycerol temperature was > 60°C.

###  Finishing

 After curing, the denture was finished and polished, and was subsequently delivered after clinical adjustments, after which postinsertion instructions were given along with denture hygiene and maintenance information.

###  Evaluation of Oral Health-related Quality of Life (OHRQoL)

**Figure 4:**
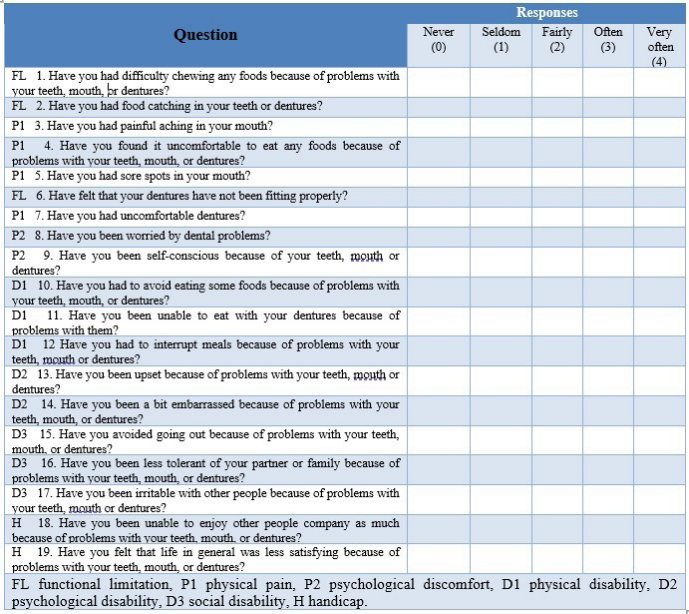
Questionnaire form

 The patients’ oral health-related quality of life (OHRQoL) was assessed using the OHIP-EDENT (Oral Health Impact Profile for Edentulous Patients instrument)(Figure 4). The OHIP-EDENT comprises 19 questions and seven subscales: functional limitations, physical pain, psychological discomfort, physical disability, psychological disability, social impairment, and handicap. This tool helps detect the impact of oral health on the quality of life of prosthetic patients.^10,11^ The assessment is specifically tailored for edentulous individuals and comprises inquiries about various aspects, such as eating pleasure, masticatory capacity, confidence, and comfort levels while wearing the prosthesis. The instrument assesses the influence of prosthetic patients’ oral health on their quality of life before and after prosthetic delivery. The questionnaire was given to the patients in Arabic. The questionnaire included five response options. A simple score of the responses to each question produced the overall score (0 = never, 1 = rarely, 2 = fairly often, 3 = often, 4 = very often). The minimum score indicates an individual’s favorable perception of their oral health, which, in turn, leads to increased satisfaction and an enhanced quality of life. The sessions lasted no more than 15 minutes each and were conducted by the same investigator during both visits.

###  Evaluation of Chewing Efficiency

 For the assessment of chewing efficiency, a commercially available two-color (pink: watermelon flavor, TRIDENT GUM, Egyp) and (White:Mint flavor, TRIDENT GUM, Egypt) chewing gum was used. Strips were cut from both colors and stuck together, yielding a test strip measuring 30 × 18 × 3 mm from one white and one pink piece.^12^

**Figure 5:**
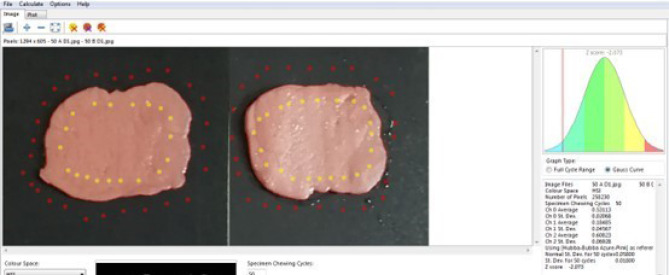
Optoelectronic analysis of chewing samples using ViewGum software

 The same investigator evaluated the participants. Each patient was asked to chew five samples of two-colored chewing gum for 5, 10, 20, 30, and 50 chewing strokes. The subjects were allowed to rest for at least 1 min between chewing trials.^13^ After rinsing the chewed gum, it was sandwiched between two sheets of transparent, rigid plastic with a 1-mm spacer between them to produce a wafer of uniform thickness. The chewed gum was trimmed to a 1 × 50 × 50-mm strip. Using a flatbed scanner, each specimen was scanned at a resolution of 500 dpi and analyzed by the operator (DH) using View Gum software(Figure 5). All the images were imported into View Gum software and automatically processed.

###  Statistical Analysis

 Data were analyzed using the Statistical Package for the Social Sciences (SPSS) for Windows (version 26.0; IBM Corp., USA). Data distribution was assessed for normality using the Shapiro–Wilk test.

 OHIP-EDENT scores demonstrated non-normal distribution; therefore, intra-subject comparisons between denture types and across evaluation intervals were performed using the Wilcoxon signed-rank test. Chewing efficiency (H-SD values) showed normal distribution; therefore, comparisons between conventional and 3D-printed dentures were performed using the paired t-test. Changes over time were analyzed using repeated-measures analysis where applicable. The level of statistical significance was set at P ≤ 0.05.

## Results

###  Patients’ Oral Health-related Quality of Life (OHRQoL)

 At T_0_, overall patient satisfaction (lower OHIP-EDENT score indicating better quality of life) was slightly better with conventional dentures than with 3D-printed dentures (12.5 ± 4.0 vs. 14.0 ± 1.4). The handicap domain scored 0 for both denture types. However, a comparison between the two denture types revealed no statistically significant differences in any domain or in the overall score (*P* > 0.05).

 At T_3_, overall satisfaction was slightly better with the 3D-printed denture compared to the conventional denture (2.5 ± 1.3 vs. 3.0 ± 1.4); however, these differences were not statistically significant (*P* > 0.05) (Table 1).

**Table 1:** Comparison of overall OHIP-EDENT scores between conventional and 3D-printed dentures at T_0_, T_3_, and T_6_

**Evaluation time point**	**Conventional (Mean±SD)**	**3D-printed (Mean±SD)**	*P ** value**
T_0_	12.5 ± 4.0	14.0 ± 1.4	0.882
T_3_	3.0 ± 1.4	2.5 ± 1.3	0.549
T_6_	0.3 ± 0.5	0.8 ± 0.5	0.186

*Wilcoxon signed-rank test used for intergroup comparisons

 A significant improvement in OHIP-EDENT scores was observed from pre-treatment to T_6_ for both denture types. For conventional dentures, the overall score decreased from 23.5 ± 4.2 pre-treatment to 0.3 ± 0.5 at T_6_ (*P* < 0.05) (Table 1). Similarly, for 3D-printed dentures, the overall score decreased from 23.5 ± 4.2 before treatment to 0.8 ± 0.5 at T_6_ (*P* < 0.05) (Table 2). Significant improvements were observed across all domains for both denture types.

**Table 2:** Comparison of OHIP-EDENT scores for conventional and 3D-printed dentures (before treatment vs. T_6_)

**Domain**	**Conventional pre-treatment (Mean±SD)**	**Conventional T**_6_ **(Mean±SD)**	* ***P *** **value**	**3D-printed pre-treatment ** **(Mean±SD)**	**3D-printed T**_6_ **(Mean±SD)**	* ***P*** ** value**
Functional limitation	3.8 ± 0.5	0.0 ± 0.0	0.011	3.8 ± 0.5	0.5 ± 0.6	0.017
Physical pain	6.3 ± 1.7	0.0 ± 0.0	0.014	6.3 ± 1.7	0.0 ± 0.0	0.014
Psychological discomfort	1.8 ± 1.0	0.0 ± 0.0	0.013	1.8 ± 1.0	0.0 ± 0.0	0.013
Physical disability	6.3 ± 0.5	0.3 ± 0.5	0.015	6.3 ± 0.5	0.3 ± 0.5	0.015
Psychological disability	2.5 ± 1.0	0.0 ± 0.0	0.011	2.5 ± 1.0	0.0 ± 0.0	0.011
Social disability	1.8 ± 0.5	0.0 ± 0.0	0.011	1.8 ± 0.5	0.0 ± 0.0	0.011
Handicap	1.3 ± 0.5	0.0 ± 0.0	0.011	1.3 ± 0.5	0.0 ± 0.0	0.011
Overall	23.5 ± 4.2	0.3 ± 0.5	0.018	23.5 ± 4.2	0.8 ± 0.5	0.018

*Wilcoxon signed-rank test

###  Chewing Efficiency

 For the conventional denture, the H-SD of the chewing test decreased progressively with increasing chewing cycles. At T_0_, H-SD decreased from 0.207 ± 0.018 at 5 cycles to 0.044 ± 0.030 at 50 cycles. A similar trend was observed at all follow-up intervals.

 For the 3D-printed denture, H-SD values also decreased with increasing chewing cycles and follow-up intervals. The highest value was recorded at T_0_ (0.215 ± 0.036 at 10 cycles), while the lowest value was observed at T_6_ (0.047 ± 0.004 at 30 cycles).

 A comparison between conventional and 3D-printed dentures at each chewing cycle revealed no statistically significant differences (*P* > 0.05) (Table 3). Although slightly higher H-SD values were observed for 3D-printed dentures at most chewing cycles (except at 30 cycles), these differences were not statistically significant.

**Table 3:** Comparison between conventional and 3D-printed dentures at different chewing cycles

**Cycle**	**Denture**	**Mean±SD**	**Mean difference**	* ***P*** ** value**
5 cycles	Conventional	0.183 ± 0.045	-0.007	0.716
	3D-printed	0.190 ± 0.050		
10 cycles	Conventional	0.133 ± 0.067	-0.013	0.654
	3D-printed	0.145 ± 0.072		
20 cycles	Conventional	0.090 ± 0.059	-0.016	0.561
	3D-printed	0.105 ± 0.071		
30 cycles	Conventional	0.077 ± 0.057	0.004	0.849
	3D-printed	0.073 ± 0.046		
50 cycles	Conventional	0.054 ± 0.027	-0.006	0.601
	3D-printed	0.060 ± 0.023		
*Paired t-test				

 No statistically significant changes in H-SD values were observed for conventional dentures (*P* = 0.330) or for 3D-printed dentures (*P* = 0.354) over time. However, a gradual reduction in H-SD values from T_0_ to T_6_ was noted for both denture types.

## Discussion

 Given that prior denture use experience is believed to have a significant impact on patient satisfaction,^14^ this study included patients with no prior denture experience.

 While prior research has not identified a significant difference in satisfaction levels between males and females,^15,16^ other studies have shown that males report higher satisfaction.^17,18^ It is hypothesised that females are more susceptible than males to concerns about their appearance, including the aesthetics of their teeth and facial profile, which could impact their level of satisfaction.

 Alves et al^19^ postulate that gender could potentially serve as a risk factor in relation to the masticatory performance in complete denture wearers. Leles et al,^20^ showed that females performed better than males. However, prior research has suggested no difference in chewing efficiency between the genders, although maximum biting force is higher in men than in women.^21-23^

 To reduce inter-patient variability in chewing efficiency and patients’ oral health-related quality of life(e.g., age, gender, muscle activity, neuromuscular control, and anatomical considerations), this study employed a crossover design in which each patient served as their own control.

###  Patients’ Oral Health-related Quality of Life (OHRQoL)

 Patient satisfaction and the impact of treatment on patients’ oral health-related quality of life are essential objectives for edentulous patient rehabilitation.^24^ Patient satisfaction depends on a multitude of factors, including the patient’s appearance and speech-related preferences, as well as chewing, stability, and comfort. Patient-reported outcome data are needed to assess the definitive outcome of dental prostheses. Treatment efficacy should be evaluated based on the patient’s subjective assessment of treatment success, as opposed to clinical evaluations, according to Heydecke et al.^25^

 Satisfaction with complete dentures is influenced by multiple factors, including biological and technical considerations that are taken into account during fabrication. Age, gender, education level, socioeconomic status, marital status, and psychological factors are additional determinants.^26^

 This study used an intra-patient study design that permits the standardization of patient variables. In this study, the patients were assessed at a pre-treatment visit and at two post-treatment visits (at 3 and 6 months). The study employed the OHIP-EDENT, a 19-item instrument comprising seven subscales: handicap, physical pain, psychological discomfort, functional limitation, and psychological disability. Each denture was used for 3 months, and the most common follow-up period after complete denture insertion was 2–3 months to allow patients to adjust to their prostheses.^27-29^

 At T_0_ and T_6_, the patients were more satisfied with conventional dentures than with 3D-printed dentures. However, comparisons between the two types of dentures revealed no significant differences across any domains during the evaluation periods. The greater satisfaction with conventional dentures at T_0_ and T_6_ may be attributable to varying fabrication methods. Although PMMA shrinks upon polymerization and its mechanical properties deteriorate over time,^30^ studies have demonstrated that conventional CDs have physical properties superior to those of 3D-printed CDs.^31,32^ These results concur with those of Ohara et al.^33^ Previous studies concluded that heat-cured PMMA exhibited superior flexural, bond, and impact strengths; in contrast, 3D-printed resins exhibited high surface roughness and porosity.^34,35^

 Regarding patients’ aesthetic preferences, Inokoshi et al^36^ reported that conventional denture fabrication is preferred over 3D printing due to its significantly superior aesthetics and stability. Compared with conventional heat-polymerized, compression-molded, and CAD/CAM-milled denture resins, 3D-printed denture resins exhibited the greatest color change.^37^

 Furthermore, artificial tooth CDs are made from a color-gradient-displaying, hard resin. These artificial teeth have a greater visual resemblance to natural teeth, whereas 3D-printed artificial teeth are comparable to resin teeth in terms of physical properties but have a single-color tone and are more easily discoloured.^38^

 Additionally, patient satisfaction improved over time. A comparison of the OHIP-EDENT scores before treatment and at T_6_ revealed significant differences in all domains. Neuromuscular adaptation may account for the gradual increase in patient satisfaction over time.

###  Chewing Efficiency

 The evaluation of chewing efficiency was conducted by scanning and digitally assessing two-colour chewing gum (mixing ability test) because it offers several advantages over the sieving method, including reduced time required to process chewed artificial test food samples, cost-effectiveness, and ease of application.^39^

 Regarding the conventional denture in this study, the H-SD of the chewing test decreased gradually with increasing chewing cycles for all times. For 3D-printed dentures, the records also varied across cycles and times, with the lowest value at 30 cycles at T_6_ and the highest at 10 cycles at T_0_. A decreased H-SD indicates increased mixing ability and a more homogenous color of the gum as mastication progresses.^40^

 Considering chewing efficiency, the comparison between conventional and 3D-printed dentures revealed no significant differences for all chewing cycles. This may be because the occlusal surface of the 3D-printed denture was replicated by scanning the conventional denture to standardize the size, alignment, and form of the teeth. Additionally, the intra-patient study design permits the standardization of patient variables, such as biting force, which can influence chewing efficiency. However, the 3D-printed dentures showed higher H-SD values (lower mixing ability) than the conventional dentures at all chewing cycles except at 30 cycles. The increased chewing efficiency of conventional complete dentures may be attributable to the lower hardness and increased flexure of 3D-printed resins compared to those of conventional materials. Consistent with these findings, after 3 months of adaptation, conventional complete dentures reportedly showed better chewing efficiency than 3D-printed complete dentures.^41^ Moreover, a significant increase in muscular activity measured by EMG was reported for conventional complete dentures compared to printed dentures. This difference may be due to the superior retention and stability of the conventional complete dentures, as well as their superior flexural strength.^42-43^

 There were no significant differences in H-SD over time for either the conventional or the 3D-printed denture. However, the H-SD decreased gradually from T_0_ to T_6_ for each denture type, indicating an improvement in masticatory efficiency over time. This improvement in masticatory function with both conventional and 3D-printed complete dentures could be due to increased adaptation and subsequent settling of the denture, which depends on tissue adaptation to the fitting surface, border seal, oral fluid viscosity and film thickness, and denture-bearing tissue resiliency.

## Limitations

 This study was limited by its short evaluation and washout periods. The implementation of a long formal washout period would have necessitated a prolonged period during which the denture was not used. However, there was no statistically significant difference between the two sets of dentures within the patient groups, indicating that the carryover effect was minimal. In addition, intrinsic material properties of the 3D-printed resin (e.g., hardness and surface roughness) were not investigated, which may influence long-term clinical performance. Finally, the relatively small sample size may limit the generalizability of the findings despite the use of a crossover design.

## Conclusion

 Complete dentures produced via 3D printing with dimethacrylate-based resins may serve as a viable alternative to conventionally manufactured complete dentures in terms of patients’ oral health-related quality of lifeand chewing efficiency.

## Competing Interests

 The authors declare that they have no known financial conflicts or personal relationships that could have influenced the work reported in this paper.

## Ethical Approval

 The patients were fully informed about the purpose and the procedures of this study and signed written consent. The study protocol received approval from the Ethics Committee of the Faculty(approval no. A04080120) and was registered at ClinicalTrials.gov (ClinicalTrials.gov Identifier: NCT06103019) (26/10/2023).

## Supplementary File

Supplementary file contains Figure S1.

## References

[1] Fenlon MR, Sherriff M (2008). An investigation of factors influencing patients’ satisfaction with new complete dentures using structural equation modelling. J Dent.

[2] Saponaro PC, Yilmaz B, Johnston W, Heshmati RH, McGlumphy EA (2016). Evaluation of patient experience and satisfaction with CAD-CAM-fabricated complete dentures: A retrospective survey study. J Prosthet Dent.

[3] Wimmer T, Gallus K, Eichberger M, Stawarczyk B (2016). Complete denture fabrication supported by CAD/CAM. J Prosthet Dent.

[4] Bidra AS, Taylor TD, Agar JR (2013). Computer-aided technology for fabricating complete dentures: systematic review of historical background, current status, and future perspectives. J Prosthet Dent.

[5] Al-Fouzan AF, Al-Mejrad LA, Albarrag AM (2017). Adherence of Candida to complete denture surfaces in vitro: A comparison of conventional and CAD/CAM complete dentures. J Adv Prosthodont.

[6] Zembic A, Wismeijer D (2014). Patient-reported outcomes of maxillary implant-supported overdentures compared with conventional dentures. Clin Oral Implants Res.

[7] Almufleh B, Emami E, Alageel O, de Melo F, Seng F, Caron E, et al. Patient satisfaction with laser-sintered removable partial dentures: A crossover pilot clinical trial. J Prosthet Dent 2018;119(4):560–7.e1. doi: 10.1016/j.prosdent.2017.04.021. 28709680

[8] Barazanchi A, Li KC, Al-Amleh B, Lyons K, Waddell JN (2017). Additive Technology: Update on Current Materials and Applications in Dentistry. J Prosthodont.

[9] Bae EJ, Jeong ID, Kim WC, Kim JH (2017). A comparative study of additive and subtractive manufacturing for dental restorations. J Prosthet Dent.

[10] Shrestha B, Niraula SR, Parajuli PK, Suwal P, Singh RK (2018). Reliability and Validity of a Nepalese Version of the Oral Health Impact Profile for Edentulous Subjects. J Prosthodont.

[11] Zani SR, Rivaldo EG, Frasca LC, Caye LF (2009). Oral health impact profile and prosthetic condition in edentulous patients rehabilitated with implant-supported overdentures and fixed prostheses. J Oral Sci.

[12] Halazonetis DJ, Schimmel M, Antonarakis GS, Christou P (2013). Novel software for quantitative evaluation and graphical representation of masticatory efficiency. J Oral Rehabil.

[13] Ragheb N, Ibrahim W (2021). Biting Force and chewing efficiency of Conventional and CAD/CAM complete dentures A Cross-over Study. Egyptian Dental Journal.

[14] De Lucena SC, Gomes SG, Da Silva WJ, Del Bel Cury AA (2011). Patients’ satisfaction and functional assessment of existing complete dentures: correlation with objective masticatory function. J Oral Rehabil.

[15] McCunniff M, Liu W, Dawson D, Marchini L (2017). Patients’ esthetic expectations and satisfaction with complete dentures. J Prosthet Dent.

[16] Zou Y, Zhan D (2015). Patients’ expectation and satisfaction with complete denture before and after the therapy. Vojnosanit Pregl.

[17] Carlsson GE, Johansson A, Johansson AK, Ordell S, Ekbäck G, Unell L (2008). Attitudes toward dental appearance in 50- and 60-Year-old subjects living in Sweden. J Esthet Restor Dent.

[18] Pan S, Awad M, Thomason JM, Dufresne E, Kobayashi T, Kimoto S (2008). Sex differences in denture satisfaction. J Dent.

[19] Alves CP, Munhoz MFV, Oliveira Nascimento GM, Nícoli GA, Paleari AG, Camargos GV (2019). The Influence of Age, Gender, Mandibular Bone Height, Previous Experience with Prostheses, and Fabrication Methods on Masticatory Performance of Complete Denture Wearers. J Prosthodont.

[20] Leles CR, Oliveira TMC, de Araújo SC, Nogueira TE, Schimmel M (2019). Individual factors associated with masticatory performance of complete denture wearers: A cross-sectional study. J Oral Rehabil.

[21] Schimmel M, Christou P, Miyazaki H, Halazonetis D, Herrmann FR, Müller F (2015). A novel colourimetric technique to assess chewing function using two-coloured specimens: Validation and application. J Dent.

[22] Ikebe K, Matsuda K, Morii K, Furuya-Yoshinaka M, Nokubi T, Renner RP (2006). Association of masticatory performance with age, posterior occlusal contacts, occlusal force, and salivary flow in older adults. Int J Prosthodont.

[23] Enkling N, Saftig M, Worni A, Mericske-Stern R, Schimmel M (2017). Chewing efficiency, bite force and oral health-related quality of life with narrow diameter implants - a prospective clinical study: results after one year. Clin Oral Implants Res.

[24] Lee CJ, Bok SB, Bae JY, Lee HH (2010). Comparative adaptation accuracy of acrylic denture bases evaluated by two different methods. Dent Mater J.

[25] Heydecke G, Locker D, Awad MA, Lund JP, Feine JS (2003). Oral and general health-related quality of life with conventional and implant dentures. Community Dent Oral Epidemiol.

[26] Singh BP, Pradhan KN, Tripathi A, Tua R, Tripathi S (2012). Effect of sociodemographic variables on complete denture satisfaction. J Adv Prosthodont.

[27] Awad MA, Lund JP, Shapiro SH, Locker D, Klemetti E, Chehade A (2003). Oral health status and treatment satisfaction with mandibular implant overdentures and conventional dentures: a randomized clinical trial in a senior population. Int J Prosthodont.

[28] Cardoso RG, Melo LA, Barbosa GA, Calderon PD, Germano AR, Mestriner WJ (2016). Impact of mandibular conventional denture and overdenture on quality of life and masticatory efficiency. Braz Oral Res.

[29] Seenivasan MK, Banu F, Inbarajan A, Natarajan P, Natarajan S, Anand Kumar V (2019). The Effect of Complete Dentures on the Quality of Life of Edentulous Patients in the South Indian Population Based on Gender and Systemic Disease. Cureus.

[30] Akin H, Tugut F, Polat ZA (2015). In vitro comparison of the cytotoxicity and water sorption of two different denture base systems. J Prosthodont.

[31] Srinivasan M, Gjengedal H, Cattani-Lorente M, Moussa M, Durual S, Schimmel M (2018). CAD/CAM milled complete removable dental prostheses: An in vitro evaluation of biocompatibility, mechanical properties, and surface roughness. Dent Mater J.

[32] Kalberer N, Mehl A, Schimmel M, Müller F, Srinivasan M (2019). CAD-CAM milled versus rapidly prototyped (3D-printed) complete dentures: An in vitro evaluation of trueness. J Prosthet Dent.

[33] Ohara K, Isshiki Y, Hoshi N, Ohno A, Kawanishi N, Nagashima S (2022). Patient satisfaction with conventional dentures vs digital dentures fabricated using 3D-printing: A randomized crossover trial. J Prosthodont Res.

[34] Al-Dulaijan YA, Alsulaimi L, Alotaibi R, Alboainain A, Alalawi H, Alshehri S (2022). Comparative Evaluation of Surface Roughness and Hardness of 3D Printed Resins. Materials (Basel).

[35] Dimitrova M, Corsalini M, Kazakova R, Vlahova A, Chuchulska B, Barile G (2022). Comparison between Conventional PMMA and 3D Printed Resins for Denture Bases: A Narrative Review. Journal of Composites Science.

[36] Inokoshi M, Kanazawa M, Minakuchi S (2012). Evaluation of a complete denture trial method applying rapid prototyping. Dent Mater J.

[37] Gruber S, Kamnoedboon P, Özcan M, Srinivasan M (2021). CAD/CAM Complete Denture Resins: An In Vitro Evaluation of Color Stability. J Prosthodont.

[38] Cha HS, Park JM, Kim TH, Lee JH (2020). Wear resistance of 3D-printed denture tooth resin opposing zirconia and metal antagonists. J Prosthet Dent.

[39] Jasser E, Salami Z, El Hage F, Makzoumé J, Boulos PJ (2020). Masticatory Efficiency in Implant-Supported Fixed Complete Dentures Compared with Conventional Dentures: A Randomized Clinical Trial by Color-Mixing Analysis Test. Int J Oral Maxillofac Implants.

[40] Tournier C, Grass M, Septier C, Bertrand D, Salles C (2014). The impact of mastication, salivation and food bolus formation on salt release during bread consumption. Food Funct.

[41] El Mallwany MS, Agamy PDT, Ismail AA (2023). Effect of Three-Dimensional (3d)-Printed Complete Dentures Versus Conventional Complete Dentures on Chewing Efficiency. ECB.

[42] Anadioti E, Musharbash L, Blatz MB, Papavasiliou G, Kamposiora P (2020). 3D printed complete removable dental prostheses: a narrative review. BMC Oral Health.

[43] Prpić V, Schauperl Z, Ćatić A, Dulčić N, Čimić S (2020). Comparison of Mechanical Properties of 3D-Printed, CAD/CAM, and Conventional Denture Base Materials. J Prosthodont.

