# Supplementary figures and images for: Comparison of patients’ oral health-related quality of life and chewing efficiency between conventional and 3D-printed complete dentures: A crossover clinical trial
Source: J Dent Res Dent Clin Dent Prospects. 2025 Dec 31;19(4):233–41. doi: 10.34172/joddd.025.44430 (PMC13179461; doi:10.34172/joddd.025.44430)

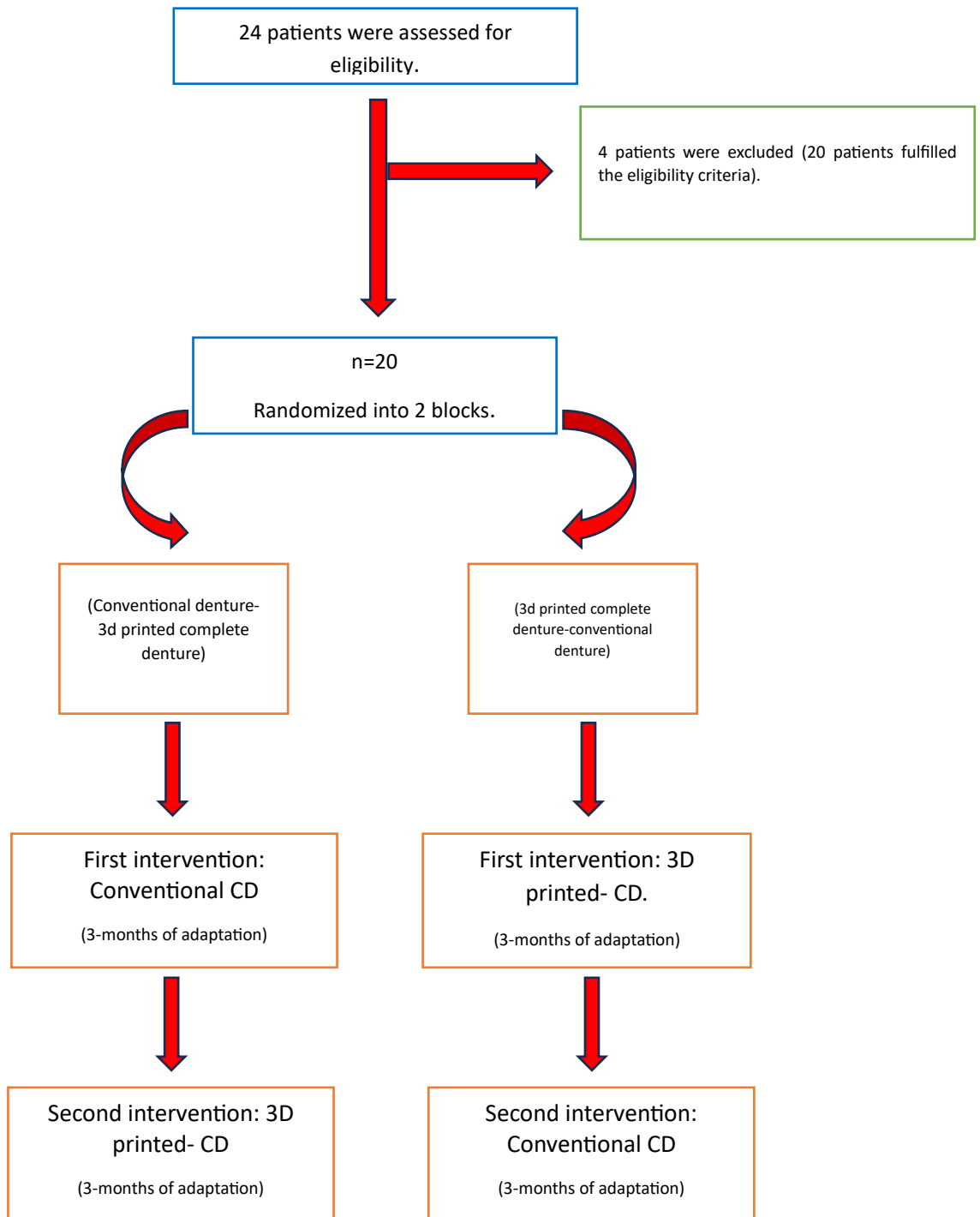

Participants flow diagram.

Supplement: Supplementary file 1 — Supplementary file contains Figure S1. [file joddd-19-233-s001.pdf]
